# Chromosome-level haplotype-resolved genome assemblies and annotations of *Apios americana* and *Apios priceana*

**DOI:** 10.1038/s41597-026-06915-y

**Published:** 2026-02-26

**Authors:** Hyun-oh Lee, Hallie C. Wright, Brandon D. Jordan, Vikas Belamkar, Jugpreet Singh, Scott R. Kalberer, Josh Clevenger, Steven B. Cannon

**Affiliations:** 1 ORISE Fellow, USDA-ARS Corn Insects and Crop Genetics Research Unit, 819 Wallace Rd, Ames, Iowa 50011 USA; 2 HudsonAlpha Institute for Biotechnology, 601 Genome Way Northwest, Huntsville, Alabama 35806 USA; 3 Corteva Agriscience, 4050 30th Ave S, Moorhead, Minnesota 56560 USA; 4 Tropical Research and Education Center, University of Florida IFAS, Homestead, FL 33031 USA; 5 USDA – Agricultural Research Service, Corn Insects and Crop Genetics Research Unit, 819 Wallace Rd, Ames, Iowa 50011 USA

**Keywords:** Genome informatics, Plant domestication, Evolutionary genetics

## Abstract

*Apios americana* and *Apios priceana* are tuber-forming legumes native to North America with ecological value and agricultural potential. The lack of genomic resources has limited comparative studies and crop improvement for these species. Here, we report high-quality, haplotype-resolved, chromosome-level genome assemblies for both the species. The assemblies were generated from high-fidelity long-read data, and the primary assemblies were scaffolded using Omni-C chromatin conformation maps. The genome sizes of the primary haplotype assemblies were 1.53 Gb for *A. americana* and 1.85 Gb for *A. priceana*, each represented by 11 pseudochromosomes. The BUSCO completeness scores ranged from 98.6% to 99.0%. Approximately 26,000 predicted genes (30,000–33,000 predicted mRNA transcripts) were identified per haplotype in *A. americana* and *A. priceana*, respectively. Repeat annotation revealed that over 80% of both genomes consist of interspersed repetitive elements, with the most abundant being long terminal repeat (LTR) retrotransposons. These genomic resources will support trait mapping and structural variation analyses in *Apios*, and more broadly, comparative genomics within the legume family.

## Background & Summary

The legume genus *Apios* comprises seven species distributed across the Himalayas, Indochina, China, Korea, Japan, eastern Canada, and the eastern and central USA^1^. These herbaceous perennial plants produce vines that display climbing or sprawling forms of growth^2^. Two species of *Apios*, *A. americana* Medikus and *A. priceana* Robinson, are indigenous to North America^3^. The perennating organ in these Nearctic species ranges from a single, irregularly spheroidal tuber (*A. priceana*) to multiple monoliform tubers spread along the length of subterranean stolons (*A. americana*)^3^. The tubers of *A. fortunei* Maximowicz of eastern Asia are intermediate in size and number although resembling *A. americana*, whereas tubers are not produced at all by *A. carnea* (Wallich) Bentham ex Baker^3,4^. Many *Apios* species are remarkable with regards to their dual capacity to produce edible stem-tubers important to asexual reproduction and to host a nitrogen-fixing symbiosis. Due to their palatability, extended shelf life, and their relatively high protein concentration (11–14% by dry weight)^5^, they have been evaluated for use as perennial crops^6,7^. Cytogenetic studies have revealed that *Apios americana* occurs in both diploid (2n = 22) and triploid (2n = 33) forms, with the triploid type being prevalent in northern populations. Conversely, *Apios priceana* was found to be uniformly diploid^8^.

In recent years, researchers have obtained chromosome-scale genomes for notable crops within Phaseoleae, including *Glycine max*^9^, *Phaseolus vulgaris*^10^, and *Vigna unguiculata*^11^. Genome assemblies for increasing numbers of non-crop species in the Phaseoleae, such as *Amphicarpaea edgeworthii*^12^ and *Spatholobus suberectus*^13^, have contributed to a broader understanding of evolution of this clade. Phylogenetic analyses indicate that *A. americana* diverged from other species in Phaseoleae early in the clade’s evolutionary history^14^. The position of *Apios* as sister to the remainder of the main phaseoloid group (Fig. S1) is in agreement with its placement in the pastid-based phylogenies of Stefanović *et al*.^14^, Wojciechowski *et al*.^15^, and Lavin *et al*.^16^. The *Apios* genomes exhibit chromosomal similarities with those of cultivated legumes such as *G. max*, *P. vulgaris*, and various *Vigna* species – for example, they have 11 chromosomes (1n), in common with most other species in the clade^17^. It is noteworthy that *Apios* is recognized as a monophyletic group within the Phaseoloid clade, with North American species forming a distinct clade from their Asian counterparts^4^.

Several characteristics provide motivation for developing genomic resources for the genus *Apios*: the perennial crop potential of species in the genus, the developmental and anatomical features of storage-roots and tubers, and the basal phylogenetic placement within a clade containing many important crop legumes. Considering this, genomic studies have yielded a transcriptome assembly and single-nucleotide polymorphism (SNP) markers for *A. americana*, identifying among the evaluated accessions six genotypic clusters and marker-trait associations for various traits^7,14^. Phenotypic evaluation of an *A. americana* collection identified four distinct genotype clusters and high-yielding genotypes, with below-ground tuber yield positively correlated with other phenological traits^15^. The long-read genome assemblies presented here will permit much higher-resolution identification of genetic variation.

**Fig. 1:**
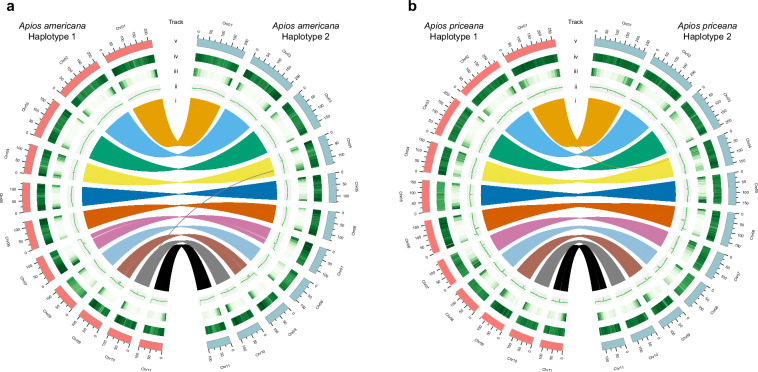
Synteny and genomic features of haplotype-resolved genomes in *Apios*. (**a**) *A. americana* haplotypes 1 and 2. (**b**) *A. priceana* haplotypes 1 and 2. Five tracks were plotted using a 1 Mb sliding window and visualized with Circos. Tracks from innermost to outermost: Track i, syntenic links between haplotypes; Track ii, GC content; Track iii, gene density; Track iv, repeat density; Track v, chromosome length.

**Fig. 2:**
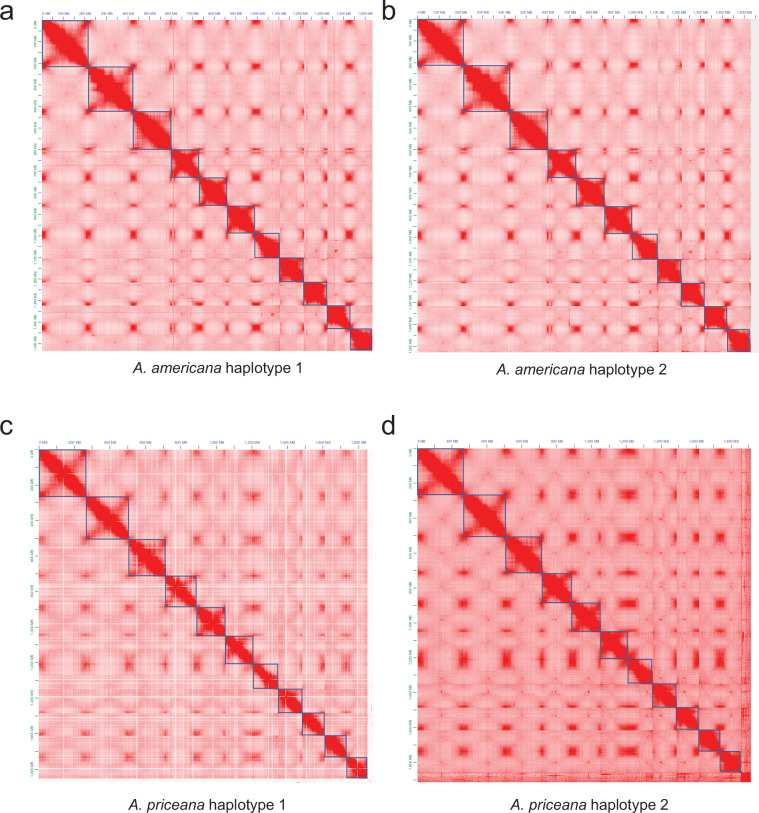
Omni-C contact maps of phased genome assemblies in *Apios americana* and *Apios priceana*. Omni-C data were used to visualize chromosome-scale interaction patterns for each haplotype assembly. (**a**) *A. americana* haplotype 1, (**b**) *A. americana* haplotype 2, (**c**) *A. priceana* haplotype 1, and (**d**) *A. priceana* haplotype 2.

In this study, we report the sequencing, assembly, and annotation of *A. americana* and *A. priceana*. The assemblies are high-quality, haplotype-resolved, chromosome-level genomes. The assembly for *A. americana* was based on long-read PacBio sequencing (181.7 Gb, ~118.8× coverage) and Omni-C chromatin conformation capture data (136.1 Gb, ~89.0× coverage). A similar strategy was employed for *A. priceana* (65.8 Gb, ~35.6× coverage long-read PacBio sequencing and 133.4 Gb, ~72.2× coverage Omni-c chromatin conformation capture data), yielding comparable coverage statistics. The final assembled genome sizes were 1.53 gigabase pairs (Gb) and 1.85 Gb for *A. americana* and *A. priceana*, respectively. Previous genome size estimates based on flow cytometry were 1.64 Gb for *A. americana* and 2.10 Gb for *A. priceana*^7^. For each of the identified haplotypes, we generated 11 pseudochromosomes, thereby anchoring over 98% of the total assembled sequence to chromosomes (Figs. 1 and 2a–d). The contig N50 sizes were 98.4–101.7 Mb for *A. americana* haplotypes and 82.3–87.7 Mb for *A. priceana* haplotypes, with GC contents of approximately 34.5% and 34.8%, respectively (Table 1). The total number of protein-coding genes predicted across the assemblies ranged from 25,667 to 27,151 (Table 2). Repeat annotation revealed that more than 80% of the genome consists of repetitive elements, predominantly long terminal repeat (LTR) retrotransposons (Table 3). The BUSCO completeness scores ranged from 98.6% to 99.0% across the four haplotypes (Table 4). The kmer completeness by Merqury demonstrated that *A. americana* haplotypes 1 and 2 were 82.2 and 81.9% complete, respectively, with an overall completeness for both haplotypes of 99.1%. The kmer completeness for the *A. priceana* haplotypes 1 and 2 were 92.8 and 92.9% complete, respectively, with an overall completeness for both haplotypes of 97.8%. The sequence error based on QV score for the *A. americana* haplotypes 1 and 2 were 38.7 and 38.6, respectively, with an overall score of 38.6. The QV score for the *A. priceana* haplotypes 1 and 2 were 56.9 and 59.4, respectively, with an overall score of 58.0.

**Table 1:** Genome Assembly Statistics of *Apios americana* and *Apios priceana*.

Genomes	*A. americana* Hap1	*A. americana* Hap2	*A. priceana* Hap1	*A. priceana* Hap2
Number of chromosomes	11	11	11	11
Assembled genome size (bp)	1,532,055,155	1,525,637,481	1,849,252,086	1,853,686,847
Min. chromosomes length (bp)	101,712,484	102,237,627	115,831,762	116,303,351
Max. chromosomes length (bp)	216,066,426	213,872,031	266,515,275	266,428,680
Min. contigs length (bp)	50,016	50,138	50,000	50,196
Max. contigs length (bp)	208,837,625	201,537,613	254,088,091	218,953,962
Average contigs length (bp)	8,659,725.0	12,374,531.4	4,906,134.3	6,656,601.2
N50 of contigs (bp)	101,712,484	101,546,880	82,267,928	87,690,639
N90 of contigs (bp)	23,020,139	24,427,629	19,250,405	16,383,316
L50 of contigs	6	6	8	8
L90 of contigs	15	17	28	25
GC Ratio (%)	34.45	34.52	34.81	34.79
bases masked (bp)	1,236,248,685	1,229,712,864	1,592,272,662	1,595,340,935
bases masked (%)	80.69%	80.60%	86.10%	86.06%

**Table 2:** Gene annotation statistics for *Apios americana* and *Apios priceana*.

Genomes	*A. americana* Hap1	*A. americana* Hap2	*A. priceana* Hap1	*A. priceana* Hap2
**Number of protein-coding genes**	25,641	25,754	26,867	26,942
**Number of transcripts (mRNAs)**	29,879	29,949	33,413	33,561
**Number of exons**	169,139	169,795	193,094	193,649
**Number of introns**	139,260	139,846	159,681	160,088
**Mean transcripts per gene**	1.2	1.2	1.2	1.2
**Mean exons per transcript**	5.7	5.7	5.8	5.8
**Mean gene length (bp)**	4,241	4,270	4,632	4,690
**Mean CDS length (bp)**	1,315	1,315	1,320	1,321
**Shortest gene (bp)**	114	120	120	120

Statistics are based on complete protein-coding models (with both start and stop codons, CDS ≥ 90 bp, and length in multiples of three).

**Table 3:** Classification of repetitive elements in the *Apios americana* and *Apios priceana* genomes.

Repeat category	*A. americana* Hap1 (bp/%)	*A. americana* Hap2 (bp/%)	*A. priceana* Hap1 (bp/%)	*A. priceana* Hap2 (bp/%)
**Total repetitive sequence**	1,236,248,685 (80.7%)	1,229,712,864 (80.6%)	1,592,272,662 (82.8%)	1,595,340,935 (83.6%)
**LTR retrotransposons (Ty1/Copia + Gypsy/DIRS1)**	465,744,395 (30.4%)	470,561,244 (30.8%)	634,490,470 (33.0%)	647,844,864 (33.9%)
**LINEs**	7,115,667 (0.46%)	4,240,789 (0.28%)	6,593,538 (0.34%)	12,312,686 (0.65%)
**SINEs**	24,783 (0.00%)	272,140 (0.02%)	6,193,155 (0.32%)	4,007,540 (0.21%)
**DNA transposons**	73,531,634 (4.8%)	68,622,745 (4.5%)	76,738,266 (4.0%)	92,484,018 (4.8%)
**Rolling-circle elements**	3,141,872 (0.21%)	3,664,012 (0.24%)	7,470,444 (0.39%)	4,387,365 (0.23%)
**Unclassified sequences**	656,806,832 (42.9%)	639,704,718 (41.9%)	790,872,301 (41.2%)	771,818,916 (40.4%)

**Table 4:** BUSCO Assessment of haplotype-resolved assemblies of *Apios americana* and *Apios priceana*.

BUSCO categories	Metric	*A. americana* Hap1	*A. americana* Hap2	*A. priceana* Hap1	*A. priceana* Hap2
**Genomes**	Complete BUSCOs	5,302 (98.8%)	5,302 (98.6%)	5,302 (98.7%)	5,302 (98.7%)
	Single-copy	5,114 (95.3%)	5,114 (95.0%)	5,114 (95.3%)	5,114 (95.2%)
	Duplicated	188 (3.5%)	188 (3.6%)	188 (3.4%)	188 (3.5%)
	Fragmented BUSCOs	11 (0.2%)	11 (0.2%)	11 (0.1%)	11 (0.1%)
	Missing BUSCOs	54 (1.0%)	54 (1.2%)	54 (1.2%)	54 (1.2%)
**Annotations**	Complete BUSCOs	5,238 (97.6%)	5,238 (97.3%)	5,238 (98.3%)	5,238 (98.4%)
	Single-copy	5,050 (94.1%)	5,050 (93.3%)	5,050 (94.7%)	5,050 (94.6%)
	Duplicated	188 (3.5%)	188 (4.0%)	188 (3.6%)	188 (3.8%)
	Fragmented BUSCOs	17 (0.3%)	17 (0.3%)	17 (0.1%)	17 (0.2%)
	Missing BUSCOs	113 (2.1%)	113 (2.4%)	113 (1.6%)	113 (1.4%)
**Total BUSCOs searched**		5,366	5,366	5,366	5,366

**Fig. 3:**
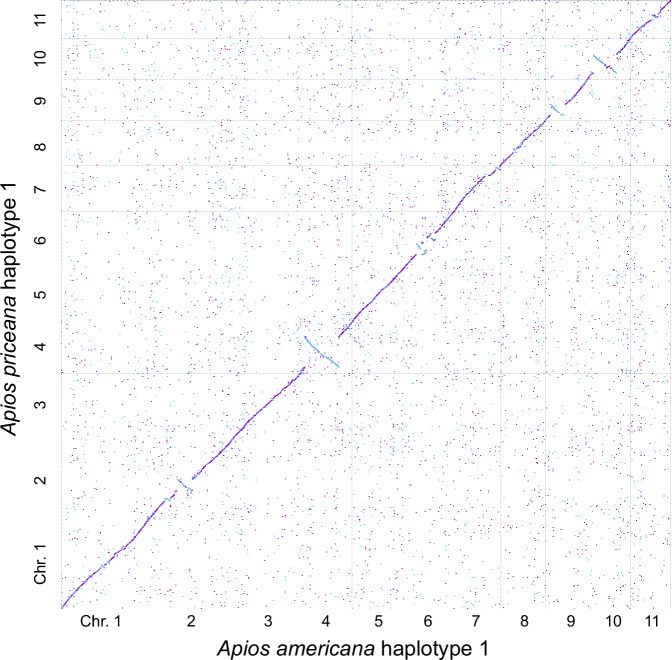
Syntenic dot plot between *Apios americana* haplotype 1 (x-axis) and *Apios priceana* haplotype 1 (y-axis). Purple dots represent forward (same-strand) syntenic blocks, while blue dots indicate inverted (reverse-strand) syntenic blocks.

Genomic comparisons among all four haplotypes (*A. americana* H1 & H2 and *A. priceana* H1 & H2) identify generally clear synteny among the 11 corresponding chromosomes, but with multi-megabase inversions on five chromosomes (Fig. 3), the largest comprising more than 82 Mbp (more than half) of chromosome 4 in the comparison between the two species. These inversions are present irrespective of the haplotype, supporting (along with the Omni-C maps) that the structural differences are features of the chromosomes rather than assembly artifacts.

The *A. priceana* genome is approximately 20% larger than that of *A. americana* (Table 1). The size differences appear due primarily to the greater repetitive content in *A. priceana*, which is approximately 86.1% repetitive compared with 80.7% in *A. americana* (Table 1). Excluding masked repetitive elements, the two genomes are approximately 94.2% identical.

Based on analysis of Ks peaks and reported fossil-calibrated divergence times in the legumes, we estimate that *A. americana* and *A. priceana* diverged ~2.23 Mya. Specifically, assuming similar average rates of change in *Apios* as in *Glycine* back to the shared papilionoid whole genome duplication approximately 58 Myr ago^9,16^, and based on a strong Ks peak of 0.025 between *A. americana* and *A. priceana* and in the *Glycine* self-comparison of 0.65 for the papilionoid WGD: 58 Mya * (0.025 Ks)/(0.65 Ks) = 2.23 Mya. See the species Ks plot at Fig. S2. A similar divergence age (2.5 Mya) results from using branch lengths in the consensus species phylogeny (Fig. S1) and an estimate of 10 Mya for the *Glycine* WGD^9^: ((10 Mya)/0.32) * 0.008 = 2.5 Mya.

**Fig. 4:**
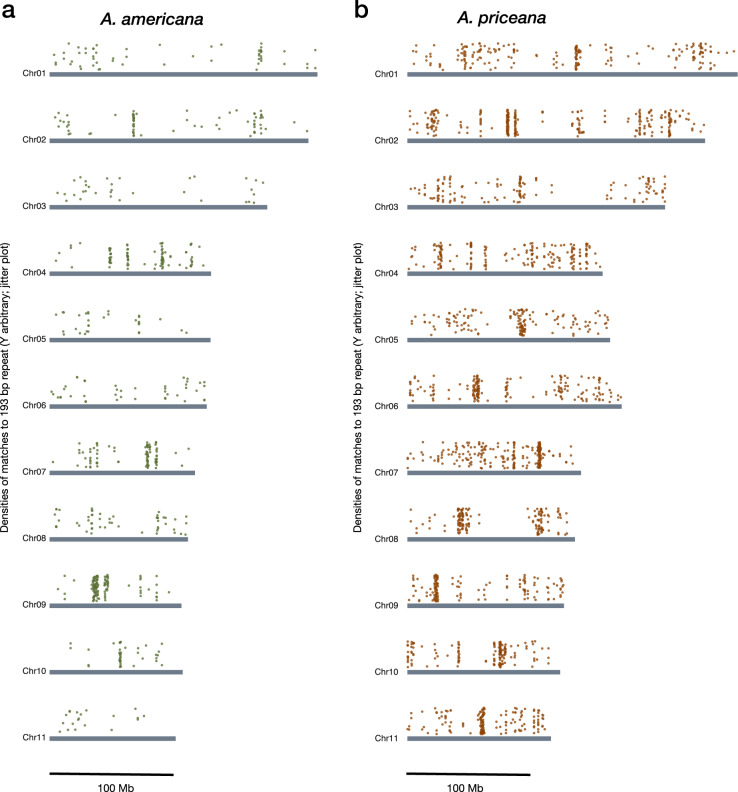
The chromosome-wise distribution of 193 bp tandem repeats (candidates for centromeric satellite repeats) in *Apios*: (**a**) *A. americana* and (**b**) *A. priceana*. Dots above each chromosome indicate densities of blastn (BLAST + with e-value of 1e-50) matches from a consensus 193 bp query. The vertical placement of the dots in the jitter plot is random; only the horizontal placement is significant.

Potential centromeric satellites were present on all eleven chromosomes of both *Apios* species (Fig. 4). The most frequent candidate centromeric repeats in both species were 193 bp, with some variation within and between the two species, but with sequence identities generally within 85%, and with a consensus sequence of AAAAATATTTAAATTATTATAACCAAGGTAGTACAGTTTTCCAAAATTATAAAANCAGTTCCACAGCATAAAGTTTCAGAATTTCAGCATACTAAATCTGTGTTAAGAAATATATTAAAAATAGTAAACGAAGTCATAAAATAACTTATTATATGTCAAATTAAAGCTTAGTATGTCTATTTTATTTTTATGT.

In *A. americana*, these satellite repeats ranged from 22 Kb to 4 Mb in length and cumulatively spanned ~14 Mb (0.92%) of the genome, with the largest arrays found on chromosomes 4, 7, and 9. In *A. priceana*, the arrays were significantly larger and more numerous, ranging from 3.8 Mb to 5 Mb and collectively covering ~39 Mb (2.11%) of the genome, with the largest arrays on chromosomes 2, 6, and 7.

These genome assemblies provide a valuable resource for comparative and evolutionary studies in legumes, particularly for understanding genome diversification, structural variation, and the genomic basis of perennial growth traits and tuber and storage-root development in Phaseoleae.

## Methods

### Plant sample and DNA preparation

*Apios americana* strain LA2127 was selected by Drs. Bill Blackmon and Mr. Berthal Reynolds in the late 1980s, from crosses derived from wild *A. americana* accessions collected from the southern U.S. (primarily Louisiana). This accession is described in Belamkar *et al*.^18^ and Belamkar *et al*.^7^.

*Apios priceana* strain MO19963523 is from a tuber grown from seed from an accession from the Missouri Botanic Garden, which in turn was grown from seed from a plant collected by Dr. George Rogers on October 13th, 1987 (1987–3021) in Lee County Mississippi.

For both *A. americana* and *A. priceana*, tissue for genomic sequencing was from etiolated young shoots from a clean tuber.

High molecular weight (HMW) DNA was extracted using the Takara Nucleobond® HMW DNA extraction kit. DNA quantity was assessed using the Qubit™ dsDNA High Sensitivity kit, and DNA quality was assessed with the Femto Pulse gDNA 165 kb analysis kit.

### PacBio and omni-C sequencing

Short DNA fragments were removed from the genomic DNA with the PacBio Short Read Eliminator (SRE) kit, and DNA was sheared using repeated pipetting cycles with the Hamilton Microlab Prep workstation. DNA was cleaned with PacBio SMRTbell® cleanup beads, and libraries were generated using the PacBio HiFi SMRTbell® library kit under manufacturer conditions. The libraries were sequenced at HudsonAlpha Institute for Biotechnology (Huntsville, AL).

Omni-C libraries were both constructed using the Dovetail® Omni-C® Kit and sequenced by the HudsonAlpha Institute for Biotechnology Genome Sequencing Center (GSC) (Huntsville, AL). A total of 450,801,658 and 441,811,210 initial read pairs for *A. americana* and *A. priceana*, respectively, were generated from the Omni-C libraries.

### Genome assembly of Apios americana and apios priceana

**Fig. 5:**
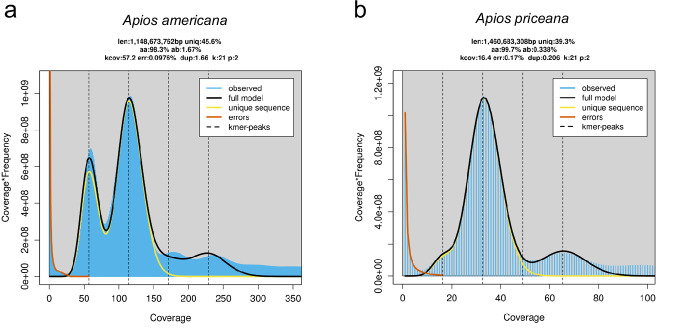
Estimation of genome size and heterozygosity using k-mer (k = 21) accessibility analysis. The k-mer frequency distributions for *Apios americana* (**a**) and *Apios priceana* (**b**) were analysed using GenomeScope. The blue areas represent the observed k-mer distributions, while the black lines indicate the best-fitting model. The yellow lines show the distribution of unique sequences and the red lines show the k-mer frequency associated with sequencing errors.

A genome survey using the long reads was conducted using JellyFish (version 2.3.1)^19^ and GenomeScope (version 2)^20^ to estimate the genome size, heterozygosity, and repeat content for both *A. americana* and *A. priceana* (Fig. 5). The k-mer distribution map with k = 21 estimated the haploid genome sizes of *A. americana* and *A. priceana* to be 1.15 Gb and 1.46 Gb, respectively, with estimated heterozygosity rates of 1.67% and 0.34%, respectively. The repeat sequence content was estimated as 54.4% and 60.7% for *A. americana* and *A. priceana*, respectively. The main assembly consisted of 14,099,941 CCS PACBIO sequences for *A. americana* and 3,502,247 for *A. priceana* (12,885.1 and 18,788.7 bp average read size, respectively), assembled using HiFiAsm (version 0.19.8) with Hi-C integration^21^ under default parameters. Contigs less than 50 Kb were filtered out. QUAST (version 5.2.0)^22^ was utilized to assess genome assembly completeness (Table 1). Kmer completeness was assessed with Merqury (version 1.3)^23^, using an initial Meryl database for each genome’s HiFi reads containing 21-mers that occur more than once. The resulting sequence was polished using RACON (1.4.3)^24^. The final assemblies were assessed once more with Omni-C data to confirm the conformation integrity was intact in the final assemblies (Fig. 2A–D).

Omni-C Samples were processed using the Dovetail® Omni-C® processing guidelines (https://github.com/dovetail-genomics/Omni-C). Briefly, Burrows Wheeler Aligner-Maximal Exact Match (BWA-MEM, version 0.7.17)^25^ was used to align the paired-end Omni-C reads to the initial Hi-C integrated-Hifiasm assemblies, and Paritools^26^ was utilized to identify ligation events, sort the read pairs, and remove PCR duplicates. The final BAM file was used as input for scaffolding the contigs as described below.

Contigs for both species and haplotypes were then oriented, ordered, and joined into chromosomes using the JUICER pipeline^27^. Chromosome-scale scaffolding was performed in 2022 with 3D-DNA^28^ (version 180922) for *A. priceana*, and chromosome-scale scaffolding was performed in 2024 for *A. Americana* with YaHS^29^ using the final BAM files generated from the Omni-C analysis as input. Contigs containing significant telomeric sequences were properly oriented in the assembly. For *A. americana*, a total of 27 contig joins were made for HAP1 and 20 contig joins were made for HAP2 to form the final assemblies, each consisting of 11 chromosomes. For *A. priceana*, a total of 89 joins were applied to the HAP1 assembly, and 128 joins for the HAP2 assembly. A total of 98.2% of the assembled sequence is contained in the chromosomes for *A. Americana* HAP1 and 99.4% of the assembled bases is contained in *A. americana* HAP2. For *A. priceana* a total of 99.8% and 99.8% of assembled bases integrated into the respective HAP1 and HAP2 chromosomes. For each assembly, chromosomes were numbered to be consistent with the first haplotype, and the resulting sequence was screened for retained vector and/or contaminants. Heterozygous SNP/indel phasing errors were identified and corrected in both *A. americana* and *A. priceana* in the aligned CCS data using Illumina reads (2 × 150, 400 bp inserts) by aligning the reads using BWA-MEM^25^ and identifying homozygous SNPs and INDELs with the GATK’s UnifiedGenotyper tool^30^.

### Repetitive elements and centromeric region characterization

Repeat annotation was performed using both *de novo* and homology-based approaches. For each genome assembly, a species-specific repeat library was first constructed using RepeatModeler^31^ (version 2.0.4) to detect long terminal repeat (LTR) retrotransposons. The resulting consensus libraries were then utilized in conjunction with a legume repeat database (legume.TE_lib_2024, comprised of 1438 repeat sequences identified in 15 diverse legume species, available at https://data.legumeinfo.org/LEGUMES/Fabaceae/repeats/TE_lib_2024.rpt.6WVT/)to annotate repetitive elements employing RepeatMasker^32^ (version 4.1.5). RepeatMasker was executed independently on each haplotype assembly of *A, americana* and *A. priceana*. The classification of repeats was conducted into the following major categories: LTR retrotransposons (Ty1/Copia and Gypsy/DIRS1), LINEs, SINEs, DNA transposons, rolling-circle elements, and unclassified sequences. The total repeat content ranged from 80.6% to 83.58% across assemblies, with LTR elements accounting for over 30% of each genome (Table 3).

Tandem repeats in A. americana and A. priceana were identified using ULTRA (version 0.1.0)^33^. Repeats with lengths ≥90 bp were evaluated for abundance, chromosomal position, and array size. Based on these characteristics, a collection of similar ~193 bp repeats were identified in both species, which had positional and clustering characteristics consistent with centromeric repeats. These were clustered, aligned, and used to derive a consensus sequence, which was used as a query in BLAST searches against both genome assemblies. BLAST hits were filtered for ≥90% sequence identity, and their genomic coordinates were merged using the bedtools merge function^34^, with a merge distance of 100 kb to define putative centromeric arrays.

To refine the locations of centromeric regions and to discriminate between ancestral and putative neocentromeres, we used RepeatObserver (https://github.com/celphin/RepeatOBserverV1). This tool calculates repeat density and coverage metrics that allow high-resolution mapping of tandem repeat clusters. For chromosomes with multiple centromeric repeat loci, the region with the highest repeat content was assumed to represent the ancestral centromere, following the reasoning that older centromeres have had more time to accumulate and homogenize satellite arrays. Conversely, smaller or spatially disjunct repeat clusters were interpreted as candidate neocentromeres.

### Genome annotation

The BRAKER3 pipeline^35^ was used to generate annotations for *A. americana* and *A. priceana* (two haplotypes for each). The pipeline was applied to repeat-masked assemblies, masked with the constructed species-specific repeat libraries that were used to soft-mask the respective genomes with RepeatMasker^32^. For the homology-based modeling using orthologous proteins, we used a set of legume-focused gene families constructed with the Pandagma gene family workflow^36^. These gene families included protein sequences of 36 legume species in 21 genera and four legume subfamilies, as well as four nonlegume outgroup genera (Table S1). For *A. americana* transcript alignments, we used 27 transcript read libraries from varied shoot, root, and tuber tissues (GenBank accessions SRR3224371-SRR3224384 (14 samples), SRR20018817, SRR20018820, SRR20018828, SRR20018831, SRR20018834, SRR20018835, SRR20018838, SRR20018841, SRR20018844, SRR20018848, SRR20018851, SRR20018854, SRR20018858). For *A. priceana* transcript alignments, we used 18 transcript read libraries from mixed shoot, root, and tuber tissues^37^. The busco_lineage parameter was set as fabales_odb10.

### Other genomic analyses

Synteny analyses and genomic alignments were conducted with MUMmer4^38^. Rates of synonymous-site changes per synonymous site (Ks values) between *A. americana*, *A. priceana*, and *G. max* were calculated using Pandagma (the “family” workflow)^36^, with the underlying Ks gene-pair calculations handled by PAML^39^.

## Data Records

*A. americana* haplotype assemblies for isolate LA2127 are registered at NCBI under Bioprojects PRJNA1251543^40^ and PRJNA1251542^41^ (Haplotypes 1 and 2 respectively), under umbrella project PRJNA1251034. The whole genome shotgun sequencing projects have been deposited at DDBJ/ENA/GenBank under the accessions JBPVNU000000000^42^ and JBPVNT000000000^43^. The respective versions described in this paper are JBPVNU000000000.1 and JBPVNT000000000.1. The raw PacBio and Omni-C data have been deposited under SRR37162738^44^ and SRR37162735^45^, respectively.

*A. priceana* haplotype assemblies for isolate MO19963523 are registered at NCBI under Bioprojects PRJNA1251038^46^ and PRJNA1252084^47^ (Haplotypes 1 and 2 respectively), under umbrella project PRJNA1252165. The whole genome shotgun sequencing projects have been deposited at DDBJ/ENA/GenBank under the accessions JBNFDG000000000^48^ and JBNFDF000000000^49^. The respective versions described in this paper are JBNFDG000000000.1 and JBNFDF000000000.1. The raw PacBio and Omni-C data have been deposited under SRR37162736^50^ and SRR37162737^51^, respectively.

Genome assemblies and annotations are also available at the Legume Information System Datastore: https://data.legumeinfo.org/Apios/americana/ and https://data.legumeinfo.org/Apios/priceana/^52^.

Annotations are also available at the Ag Data Commons under DOI 10.15482/USDA.ADC/29274848^53^.

## Technical Validation

The completeness of the haplotype-resolved genome assemblies for *A. americana* and *A. priceana* was assessed using BUSCO (version 5.4.3) with the fabales_odb10 lineage dataset (n = 5,366 orthologs). All four assemblies demonstrated high levels of completeness, with BUSCO scores ranging from 98.6% to 98.8% (Table 4). Among these, over 95% of BUSCO genes were identified as single-copy, while duplicated BUSCOs accounted for approximately 3.4–3.6%. The presence of fragmented and missing BUSCOs ranged from 1.0% to 1.2%.

The annotations also show complete BUSCO scores, with 97.3% to 98.4% recovery of 5,366 orthologs from the fabales_odb10 dataset (Table 4). Among these, 93.3% to 94.7% of BUSCO genes were identified as single-copy, while duplicated BUSCOs accounted for approximately 3.5 to 3.8%. The presence of fragmented and missing BUSCOs ranged from 1.4% to 2.4%.

## Supplementary information

Supplementary data and analysis for: Chromosome-level haplotype-resolved genome assemblies and annotations of Apios americana and Apios priceana

## Data Availability

All code used for analysis in this work was from published bioinformatics tools, with software and versions indicated under Methods.
