# Supplementary material for: Chromosome-level haplotype-resolved genome assemblies and annotations of Apios americana and Apios priceana
Source: Sci Data. 2026 Feb 26;13:544. doi: 10.1038/s41597-026-06915-y (PMC13061880; doi:10.1038/s41597-026-06915-y)
Supplement: Supplementary file 1 — Supplementary data and analysis for: Chromosome-level haplotype-resolved genome assemblies and annotations of Apios americana and Apios priceana [file 41597_2026_6915_MOESM1_ESM.docx]

**Supplementary data and analysis for: Chromosome-level haplotype-resolved genome assemblies and annotations of *Apios americana* and *Apios priceana***

**Authors**

Hyun-oh Lee^1^, Hallie C. Wright^2^, Brandon D. Jordan^1^, Vikas Belamkar^3^, Jugpreet Singh^4^, Scott R. Kalberer^5^, Josh Clevenger^2^, Steven B. Cannon^5*^

**Supplementary Information:**

Figure S1. Consensus species phylogeny for *Apios* and selected other legume species and non-legume outgroups. Page 1

Figure S2. Rates of synonymous substitutions (Ks values) in gene pairs between *Apios americana* and *Apios priceana*. Page 2

Table S1. Genomes incorporated into gene families for genome annotation. Page 3


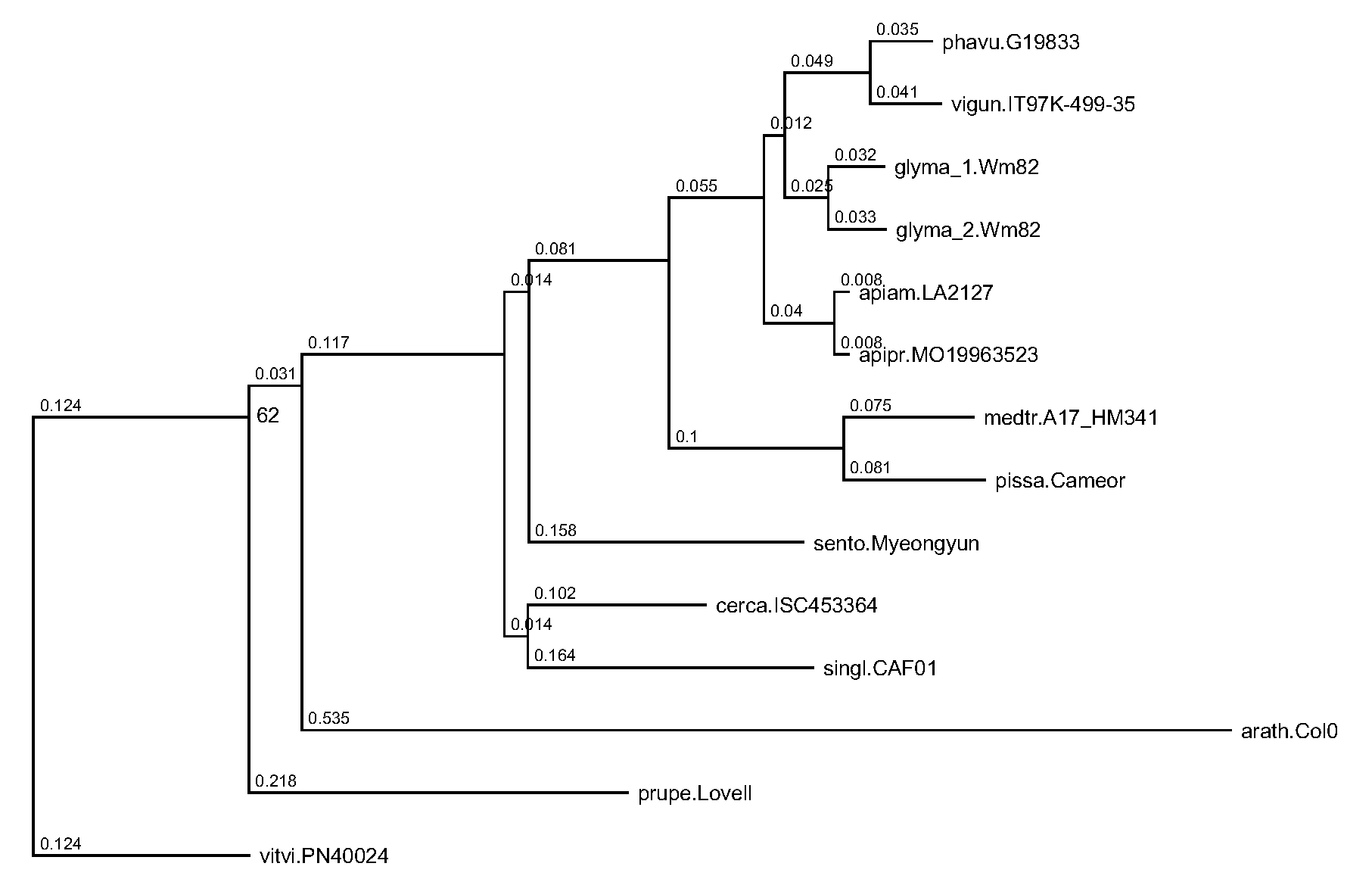


**Supplementary Figure 1.** Consensus species phylogeny for *Apios* and selected other legume species and non-legume outgroups. Species labels have the form “gensp.accession”, where gensp is composed of three letters from the genus and two from the species; and the accession is the individual used in the analysis; for example, “arath.Col0” for *Arabidopsis thaliana*, Columbia 0. The species, in order from top to bottom, are: *Phaseolus vulgaris, Vigna unguiculata, Glycine max* (two WGD-derived haplotypes), *Apios americana, Apios priceana, Medicago truncatula, Pisum sativum, Senna tomentosa, Cercis canadensis, Sindora glauca, Arabidopsis thaliana, Prunus persica, Vitis vinifera.* Branch lengths are as indicated. Bootstrap values at all nodes were 100% except at the node of *Prunus persica* and the remaining members, where support was 62%.

The consensus phylogeny was calculated from a concatenated supermatrix of the alignments of 1585 gene families in which each legume species existed in single copy, except for *Glycine* with two copies (from the *Glycine* WGD). Gene families were calculated using Pandagma (Cannon et al., 2024). The concatenated alignment was generated from all 1585 gene families, with genes and paralogs placed in consistent order. The alignment was sampled at modulo 7 (taking every seventh amino acid) to make phylogenetic calculations tractable. The resulting alignment matrix had 110,547 sites. The consensus phylogeny was then calculated using RAxML-NG (Kozlov et al., 2019), using the raxml-ng --all workflow, with model LG+FC+G8m{0.562520}.

**
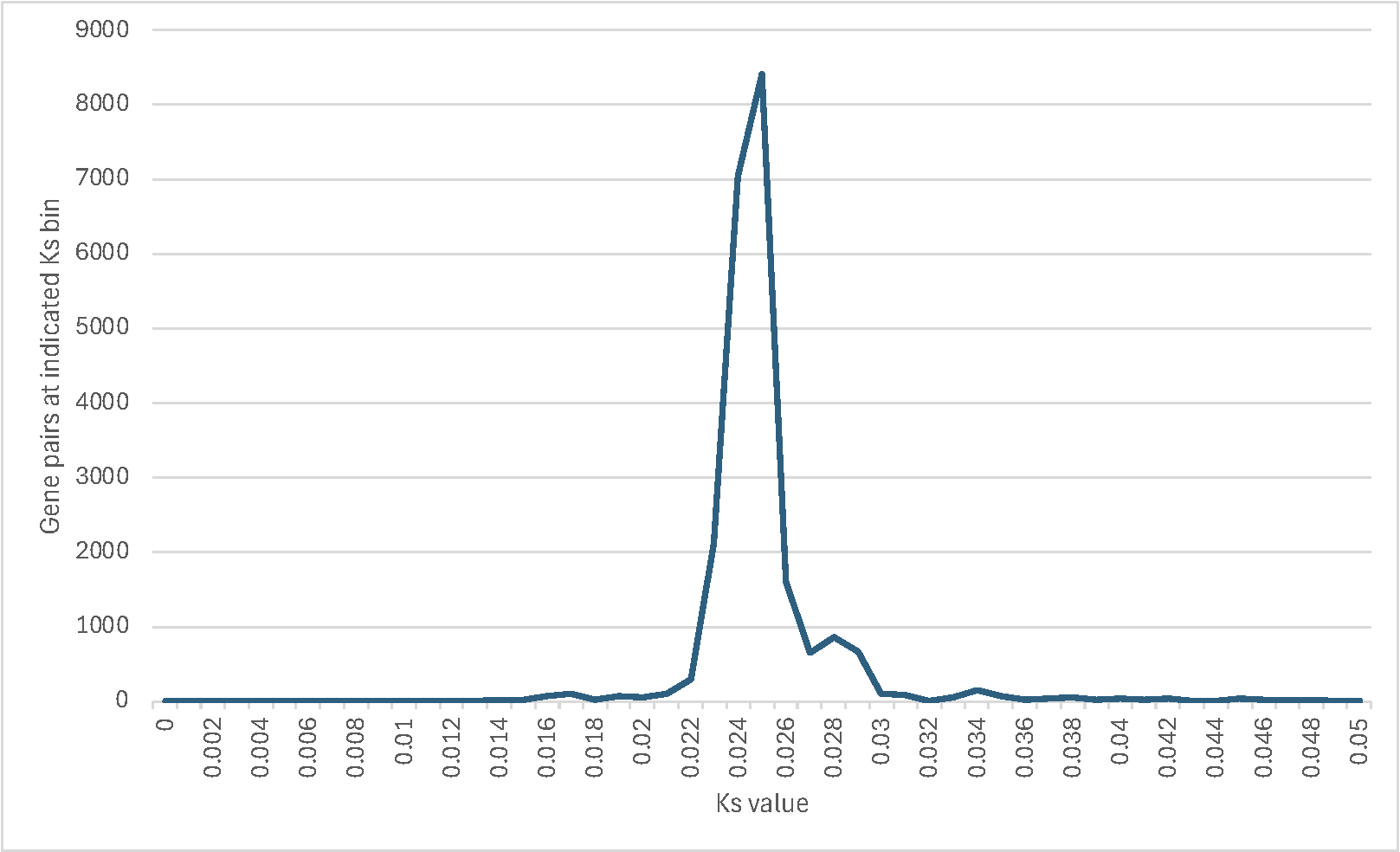
**

**Supplementary Figure 2.** Rates of synonymous substitutions (Ks values) in gene pairs between *Apios americana* and *Apios priceana*. Ks values were calculated using PAML (Yang, 2007). A median value was then calculated for all genes in a synteny block. For the purpose of calculating Ks frequencies, median values for the genes in the block were applied to all gene pairs in that block, which were in turn used to calculate genome-wide Ks-value frequency distributions. Bins are in 0.001 Ks units.

**Supplementary Table 1.** Genomes incorporated into gene families for genome annotation. For genera with multiple species, these were first incorporated into genus-level pangenes.

| **Genus** | **Species** | **Subfamily** | **Citation** | **doi** |
| --- | --- | --- | --- | --- |
| *Acacia* | *crassocarpa* | Caesalpinioideae | Massaro et al., 2023 | 10.1093/g3journal/jkad284 |
| *Aeschynomene* | *evenia* | Papilionoideae | Quilbé et al., 2021 | 10.1038/s41467-021-21094-7 |
| *Cercis* | *canadensis* | Cercidoideae | Lee et al., 2024 | 10.1101/2024.09.03.611065 |
| *Chamaecrista* | *fasciculata* | Caesalpinioideae | Lee et al., 2024 | 10.1101/2024.09.03.611065 |
| *Dalbergia* | *odorata* | Papilionoideae | Hong et al., 2020 | 10.1093/gigascience/giaa084 |
| *Lablab* | *purpureus* | Papilionoideae | Njaci et al., 2023 | 10.1038/s41467-023-37489-7 |
| *Lens* | *culinaris* | Papilionoideae | Ramsay et al., 2021 | 10.1101/2021.07.23.453237 |
| *Lotus* | *japonicus* | Papilionoideae | Sato et al., 2008 | 10.1093/dnares/dsn008 |
| *Lupinus* | *albus* | Papilionoideae | Hufnagel et al., 2020 | 10.1038/s41467-019-14197-9 |
| *Phanera* | *championii* | Cercidoideae | Lu et al., 2024 | 10.1111/tpj.16620 |
| *Pisum* | *sativum* | Papilionoideae | Kreplak et al., 2019 | 10.1038/s41588-019-0480-1 |
| *Senna* | *tomentosa* | Papilionoideae | Kang et al., 2020 | 10.1038/s41467-020-19681-1 |
| *Sindora* | *glauca* | Detarioideae | Yu et al., 2022 | 10.3389/fpls.2021.794830 |
| *Trifolium* | *pratense* | Papilionoideae | De Vega et al., 2015 | 10.1038/srep17394 |
| *Vicia* | *faba* | Papilionoideae | Jayakodi et al., 2023 | 10.1038/s41586-023-05791-5 |
| *Arachis* | *hypogaea* | Papilionoideae | Bertioli et al., 2019 | 10.1038/s41588-019-0405-z |
| *Arachis* | *stenosperma* | Papilionoideae | Bertioli et al., 2019 | 10.1038/s41588-019-0405-z |
| *Arachis* | *duranensis* | Papilionoideae | Bertioli et al., 2016 | 10.1038/ng.3517 |
| *Arachis* | *ipaensis* | Papilionoideae | Bertioli et al., 2016 | 10.1038/ng.3517 |
| *Cicer* | *arietinum* | Papilionoideae | Garg et al., 2021 | 10.1016/j.jare.2021.10.009 |
| *Cicer* | *echinospermum* | Papilionoideae | Cook et al., 2022 | GenBank GCA_002896215.2 |
| *Cicer* | *reticulatum* | Papilionoideae | Cook et al., 2022 | GenBank GCA_002896235.1 |
| *Glycine* | *max* | Papilionoideae | Schmutz et al., 2010 | 10.1038/nature08670 |
| *Glycine* | *soja* | Papilionoideae | Xie et al., 2019 | 10.1038/s41467-019-09142-9 |
| *Glycine* | *cyrtoloba* | Papilionoideae | Zhuang et al., 2022 | 10.1038/s41477-022-01102-4 |
| *Glycine* | *dolichocarpaD3* | Papilionoideae | Zhuang et al., 2022 | 10.1038/s41477-022-01102-4 |
| *Glycine* | *tomentella-D3* | Papilionoideae | Zhuang et al., 2022 | 10.1038/s41477-022-01102-4 |
| *Glycine* | *falcata* | Papilionoideae | Zhuang et al., 2022 | 10.1038/s41477-022-01102-4 |
| *Glycine* | *stenophita* | Papilionoideae | Zhuang et al., 2022 | 10.1038/s41477-022-01102-4 |
| *Glycine* | *syndetika* | Papilionoideae | Zhuang et al., 2022 | 10.1038/s41477-022-01102-4 |
| *Medicago* | *truncatula* | Papilionoideae | Tang et al., 2014 | 10.1186/1471-2164-15-312 |
| *Medicago* | *sativa* | Papilionoideae | Chen et al., 2020 | 10.1038/s41467-020-16338-x |
| *Phaseolus* | *acutifolius* | Papilionoideae | Moghaddam et al., 2021 | 10.1038/s41467-021-22858-x |
| *Phaseolus* | *lunatus* | Papilionoideae | Garcia et al., 2021 | 10.1038/s41467-021-20921-1 |
| *Phaseolus* | *vulgaris* | Papilionoideae | Schmutz et al., 2014 | 10.1038/ng.3008 |
| *Vigna* | *angularis* | Papilionoideae | Sakai et al., 2015 | 10.1038/srep16780 |
| *Vigna* | *radiata* | Papilionoideae | Ha et al., 2021 | 10.1002/tpg2.20121 |
| *Quillaja* | *saponaria* | Quillajaceae | Reed et al., 2023 | 10.1126/science.adf3727 |
| *Arabidopsis* | *thaliana* | Brassicaceae | Cheng et al., 2017 | 10.1111/tpj.13415 |
| *Prunus* | *persica* | Rosaceae | Verde et al., 2017 | 10.1186/s12864-017-3606-9 |
| *Vitis* | *vinifera* | Vitaceae | The French–Italian Public Consortium for Grapevine Genome Characterization, 2007 | 10.1038/nature06148 |

**References**

Cannon, S.B., Lee, H.-O., Weeks, N.T. and Berendzen, J. (2024) Pandagma: A tool for identifying pan-gene sets and gene families at desired evolutionary depths and accommodating whole genome duplications. Bioinformatics, btae526.

Kozlov, A.M., Darriba, D., Flouri, T., Morel, B. and Stamatakis, A. (2019) RAxML-NG: a fast, scalable and user-friendly tool for maximum likelihood phylogenetic inference. Bioinformatics, 35, 4453–4455.

Yang, Z. PAML 4: phylogenetic analysis by maximum likelihood. Mol. Biol. Evol. 24, 1586–1591 (2007).
